# Serum Potassium and Glucose Regulation in the ADDITION-Leicester Screening Study

**DOI:** 10.1155/2015/923749

**Published:** 2015-03-25

**Authors:** Patrice Carter, Danielle H. Bodicoat, Lauren M. Quinn, Francesco Zaccardi, David R. Webb, Kamlesh Khunti, Melanie J. Davies

**Affiliations:** ^1^University of Leicester, Diabetes Research Centre, Leicester Diabetes Centre, Leicester General Hospital, Leicester LE5 4PW, UK; ^2^Nuffield Science Bursary, University of Birmingham, Birmingham B15 2TT, UK

## Abstract

*Introduction*. Previous observational studies have shown conflicting results between plasma K^+^ concentrations and risk of type 2 diabetes. To help clarify the evidence we aimed to determine whether an association existed between serum K^+^ and glucose regulation within a UK multiethnic population. *Methods*. Participants were recruited as part of the ADDITION Leicester study, a population based screening study. Individuals from primary care between the age of 40 and 75 years if White European or 25 and 75 years if South Asian or Afro Caribbean were recruited. Tests for associations between baseline characteristics and K^+^ quartiles were conducted using linear regression models. *Results*. Data showed individuals in the lowest K^+^ quartile had significantly greater 2-hour glucose levels (0.53 mmol/L, 95% CI: 0.36 to 0.70, *P* ≤ 0.001) than those in the highest K^+^ quartile. This estimation did not change with adjustment for potential confounders. Conversely, participants in the lowest K^+^ quartile had a 0.14% lower HbA1c (95% CI −0.19 to −0.10: *P* ≤ 0.001) compared to those in the highest K^+^ quartile. *Conclusion*. This cross-sectional analysis demonstrated that lower K^+^ was associated with greater 2 hr glucose. The data supports the possibility that K^+^ may influence glucose regulation and further research is warranted.

## 1. Introduction 

The prevalence of type 2 diabetes (T2DM) is currently estimated to be 8.3% worldwide and has increased rapidly over past decades [1]. This coincides with changes in lifestyle behaviours including an increased consumption of processed foods and a decreased intake of fruit and vegetables [2]. Fruit and vegetables contain a number of potentially beneficial components, including potassium (K^+^), a major intracellular cation of the body.

Previous observational studies have investigated the relationship between serum K^+^, glucose regulation, and risk of T2DM [3, 4]. The Atherosclerosis Risk in the Communities (ARIC) study demonstrated an inverse association between serum K^+^ and fasting insulin levels, and risk of T2DM [5]; this relationship was confirmed in the TOPICS 1 study [4]. However a recent study which investigated K^+^ in individuals with impaired glucose regulation only found a significant relationship in those with established hypertension [6]. To help clarify the evidence we aimed to determine whether an association existed between serum K^+^ and glucose regulation within a UK multiethnic population; this is the first UK study to conduct analysis in a high risk population and thus extends the literature available.

## 2. Materials and Methods

Participants were recruited as part of the ADDITION Leicester study, a population based screening study that formed part of ADDITION-Europe, described in detail elsewhere [7]. All individuals were given a 75 g oral glucose load after an overnight fast and diagnosis of diabetes was made according to 1999 WHO criteria [8]. Plasma glucose was determined using the hexokinase method (Abbott laboratories, Maidenhead, UK; coefficient of variation (CV) 1.61%). HbA1c was quantified using high performance liquid chromatography on an automated DCCT aligned BioRad aligned system (BioRad laboratories, Hemel Hempstead, UK; CV < 0.1%). Serum total, HDL, LDL-cholesterol, and triglycerides were measured by enzymatic methods (Dade Behring Dimension Analyser, Newark, USA). Plasma creatinine was analysed with kinetic colorimetric methods. Serum potassium was measured using a Siemens ADVIA 2400 Clinical Chemistry System (Siemens Health Care, Germany; CV < 1%). Analyses were conducted in the pathology laboratories of the University Hospitals of Leicester, NHS Trust, UK. Anthropometric measurements were taken by trained staff following standard operating procedures.

The study was conducted according to the guidelines laid down in the Declaration of Helsinki and all procedures involving human subjects were approved by Leicestershire, Rutland, and Northamptonshire research ethics committee. Written informed consent was obtained from all subjects. The study is registered with ISCRCTN http://www.controlled-trials.com (NCT00318032).

## 3. Statistical Analysis

Baseline characteristics were summarised for the whole study population and separately by K^+^ quartiles. Data were checked for normality by visually assessing normality plots. Tests for associations between baseline characteristics and K^+^ quartiles (<4.0 mmol/L, 4.0–4.2 mmol/L, 4.3–4.4 mmol/L, and ≥4.5 mmol/L) were conducted using linear regression models: (1) unadjusted; (2) adjusted for baseline measures of average systolic and diastolic blood pressure and for Cockcroft-Gault estimated glomerular filtration rate; (3) adjusted for the previous confounders plus ethnicity, sex, age, and BMI. In sensitivity analyses we restricted the estimations in the subset of participants with normal glucose regulation at baseline (*n* = 5,249) and in those who were not taking any blood pressure medication (diuretics/thiazides, ACE-inhibitors, beta-blockers, or angiotensin-II receptor antagonists) (*n* = 5,187). Two sided *P*-value of <0.05 was considered statistically significant.

## 4. Results

From 6,749 individuals screened for diabetes we excluded those with missing K^+^ data (*n* = 154), those with K^+^ greater than 5.5 mmol/L (*n* = 62), and those with renal dysfunction (creatinine level > 1.7 mg/dL (150 *μ*mol/L) (*n* = 13), thus leaving 6,520 participants for the analyses. Of these 4,523 (69.4%) were White European and 1,629 (25.0%) were South Asian. Baseline characteristics are presented in Table 1 for the whole population and separately by K^+^ quartile. At screening 5,249 (81%) had normal glucose tolerance, 1,054 (16%) had impaired glucose regulation, and 205 (3%) were newly diagnosed with T2DM.

Participants in the lowest K^+^ quartile had significantly greater 2-hour glucose levels than those in the highest K^+^ quartile (0.53 mmol/L, 95% confidence interval (CI): 0.36 to 0.70, *P* ≤ 0.001; Tables 1 and 2). This estimation did not change with adjustment for potential confounders; model (2): 0.49 mmol/L; 95% CI: 0.29 to 0.63; *P* ≤ 0.001 and model (3): 0.49 mmol/L; 95% CI: 0.33 to 0.66; *P* ≤ 0.001. Conversely those in the lowest K^+^ quartile had a 0.14% lower HbA1c (95% CI: −0.19 to −0.10: 0. *P* ≤ 0.001) compared to those in the highest K^+^ quartile; again adjustment for confounders did not alter the association (Table 3). There was no association between K^+^ quartiles and fasting blood glucose in either the unadjusted or the adjusted models (Table 4). Sensitivity analyses restricted to participants with normal glucose regulation and excluding participants taking antihypertensive medication, including thiazides, did not change the results (data not shown).

## 5. Discussion

This cross-sectional analysis of individuals who were screened for T2DM demonstrated that lower K^+^ was associated with greater 2 hr glucose. No associations were observed between fasting plasma glucose and K^+^. In contrast those with greater K^+^ had higher HbA1c; however, although statistically significantly different, a change of 0.14% HbA1c is unlikely to be considered clinically relevant. The discrepancy between findings in 2 hr glucose and HbA1c could likely reflect the greater sensitivity of the 2 hr glucose to insulin secretion; indeed this process is controlled by ATP-sensitive potassium channels [9]. Additional subanalysis solely of those with normal glucose tolerance at baseline further showed that in otherwise healthy individuals low serum K^+^ was associated with greater 2 hr glucose, suggesting that low serum K^+^ may be implicated in the development of impaired glucose regulation. Prospective studies have previously observed that low baseline K^+^ levels are predictive of development of T2DM [4, 5]. We do not have sufficient prospective data to examine this in our study.

This study provides a unique dataset of subjects from a multiethnic population; we have conducted analysis to account for potential confounding variables and carried out sensitivity analysis to exclude those taking antihypertensive medication which may influence K^+^ concentrations. Nonetheless several limitations should be mentioned; the measurement of K^+^ was from a single blood sample at the baseline visit; however stringent laboratory conditions were in place to ensure the accuracy of samples. Information on dietary K^+^ consumption or the intake of supplements which may include K^+^ was not available. It is also important to note all levels of K^+^ in our study were within normal physiological levels. We excluded those with creatinine levels greater or equal to 1.7 mg/dL, thus attempting to exclude renal impairment as a potentially confounding factor. Indeed the population appears relatively healthy (mean blood pressure of the cohort is within the normal range), and this should be considered when comparing the data to other populations. Finally this study is cross-sectional by design; therefore we cannot determine the direction of association; however available data supports the possibility that K^+^ may influence glucose regulation and further research is warranted.

## Figures and Tables

**Table 1:** Characteristics of 6520 participants in the ADDITION-Leicester cross-sectional screening study stratified by baseline serum potassium level.

			K^+^ quartile								
Characteristic	Total Cohort (*n* = 6520)		<4.0 mmol/L (*n* = 1295)		4.0–4.2 mmol/L (*n* = 1840)		4.3–4.4 mmol/L (*n* = 1306)		≥4.5 mmol/L (*n* = 2079)		*P* for trend^a^
	*N *	%	*N *	%	*N *	%	*N *	%	*N *	%	
Female	3429	52.6	710	54.8	1084	58.9	692	53.0	943	45.4	≤0.001
Diuretics/thiazides	648	9.6	258	39.8	204	31.5	73	11.3	112	17.3	0.192
ACE-inhibitors	424	6.3	95	22.4	97	22.9	73	17.2	158	37.3	0.643
beta-blockers	619	9.2	125	20.2	154	24.9	113	18.3	226	36.5	0.630
Angiotensin-II receptor antagonists	223	3.3	58	26.0	59	26.5	35	15.7	70	31.4	0.542

	Mean	SD	Mean	SD	Mean	SD	Mean	SD	Mean	SD	

Age (years)	56.0	10.8	56.3	10.9	55.4	10.9	55.6	10.8	56.7	10.6	0.061
BMI (kg/m^2^)	28.1	5.0	28.0	5.2	28.0	5.1	27.9	4.7	28.2	5.0	0.317
Waist circumference (cm)	93.9	13.1	93.7	14.1	93.0	13.3	93.5	12.1	95.0	12.9	≤0.001

Systolic blood pressure (mmHg)	136.9	19.6	136.5	20.1	136.4	19.5	136.1	19.2	138.0	19.6	0.023
Diastolic blood pressure (mmHg)	85.4	10.6	84.9	11.0	85.3	10.5	85.0	10.3	85.9	10.6	0.022
EGFR	75.8	13.2	75.7	12.8	76.4	13.5	76.0	12.6	75.2	13.3	0.061

2-hour glucose (mmol/L)	6.0	2.4	6.4	2.4	6.1	2.4	5.9	2.3	5.9	2.4	≤0.001
Fasting blood glucose (mmol/L)	5.2	0.9	5.2	1.0	5.2	1.0	5.2	0.8	5.2	0.8	0.483
HbA1c (%)	5.7	0.6	5.6	0.6	5.7	0.6	5.7	0.5	5.8	0.6	≤0.001

K^+^ (mmol/L)	4.3	0.4	3.7	0.2	4.1	0.1	4.3	0.1	4.7	0.2	≤0.001

^a^ *P* values for trend were estimated using logistic or linear regression and test whether there is a linear trend in the outcome across potassium categories.

SD: standard deviation and eGFR: estimated glomerular filtration rate (Cockroft-Gault formula).

**Table 2:** Linear regression to determine the difference in 2-hour glucose across K^+^ quartiles.

Models	K^+^ quartiles			
	<4.0 mmol/L	4.0–4.2 mmol/L	4.3–4.4 mmol/L	≥4.5 mmol/L
Model 1^a^	0.53 (0.36 to 0.70)	0.20 (0.04 to 0.35)	−0.02 (−0.19 to 0.15)	Reference
	*P* ≤ 0.001	*P* = 0.01	*P* = 0.85	

Model 2^b^	0.49 (0.29 to 0.63)	0.17 (0.02 to 0.32)	0.02 (−0.15 to 0.19)	Reference
	*P* ≤ 0.001	*P* = 0.02	*P* = 0.80	

Model 3^c^	0.49 (0.33 to 0.66)	0.22 (0.07 to 0.37)	0.05 (−0.11 to 0.21)	Reference
	*P* ≤ 0.001	*P* = 0.003	*P* = 0.53	

Data reported as difference in 2-hour glucose (95% confidence intervals).

^a^Unadjusted.

^b^Adjusted for baseline measures of average systolic and diastolic blood pressure and for Cockcroft-Gault estimated glomerular filtration rate.

^c^Adjusted for the confounders in model 2, plus ethnicity, sex, age, and BMI.

**Table 3:** Linear regression to determine the difference in HbA1c across K^+^ quartiles.

Models	K^+^ quartiles			
	<4.0 mmol/L	4.0–4.2 mmol/L	4.3–4.4 mmol/L	≥4.5 mmol/L
Model 1^a^	−0.14 (−0.19 to −0.10)	−0.08 (−0.12 to −0.04)	−0.05 (−0.09 to −0.006)	Reference
	*P* ≤ 0.001	*P* ≤ 0.001	*P* = 0.03	

Model 2^b^	−0.15 (−0.19 to −0.11)	−0.08 (−0.12 to −0.04)	−0.04 (−0.08 to 0.001)	Reference
	*P* ≤ 0.001	*P* ≤ 0.001	*P* = 0.05	

Model 3^c^	−0.13 (−0.17 to −0.09)	−0.06 (−0.10 to −0.02)	−0.03 (−0.07 to 0.01)	Reference
	*P* ≤ 0.001	*P* = 0.002	*P* = 0.15	

Data reported as difference in HbA1c (95% confidence intervals).

^a^Unadjusted.

^b^Adjusted for baseline measures of average systolic and diastolic blood pressure and for Cockcroft-Gault estimated glomerular filtration rate.

^c^Adjusted for the confounders in model 2, plus ethnicity, sex, age, and BMI.

**Table 4:** Linear regression to determine the difference in fasting blood across K^+^ quartiles.

Models	K^+^ quartiles			
	<4.0 mmol/L	4.0–4.2 mmol/L	4.3–4.4 mmol/L	≥4.5 mmol/L
Model 1^a^	−0.01 (−0.07 to 0.05)	−0.04 (−0.09 to 0.02)	−0.06 (−0.12 to 0.006)	Reference
	*P* = 0.76	*P* = 0.19	*P* = 0.08	

Model 2^b^	−0.02 (−0.08 to 0.04)	−0.05 (−0.11 to 0.12)	−0.05 (−0.11 to 0.01)	Reference
	*P* = 0.43	*P* = 0.08	*P* = 0.12	

Model 3^c^	0.01 (−0.05 to 0.07)	−0.001 (−0.05 to 0.05)	−0.02 (−0.08 to 0.04)	Reference
	*P* = 0.75	*P* = 0.97	*P* = 0.49	

Data reported as difference in fasting blood glucose (95% confidence intervals).

^a^Unadjusted.

^b^Adjusted for baseline measures of average systolic and diastolic blood pressure and for Cockcroft-Gault estimated glomerular filtration rate.

^c^Adjusted for the confounders in model 2, plus ethnicity, sex, age, and BMI.
